# Nociception-Dependent CCL21 Induces Dorsal Root Ganglia Axonal Growth *via* CCR7-ERK Activation

**DOI:** 10.3389/fimmu.2022.880647

**Published:** 2022-07-14

**Authors:** Francina Mesquida-Veny, Sara Martínez-Torres, Jose Antonio Del Rio, Arnau Hervera

**Affiliations:** ^1^ Molecular and Cellular Neurobiotechnology, Institute for Bioengineering of Catalonia (IBEC), Barcelona, Spain; ^2^ Department of Cell Biology, Physiology and Immunology, University of Barcelona, Barcelona, Spain; ^3^ Network Centre of Biomedical Research of Neurodegenerative Diseases (CIBERNED), Institute of Health Carlos III, Ministry of Economy and Competitiveness, Madrid, Spain; ^4^ Institute of Neuroscience, University of Barcelona, Barcelona, Spain

**Keywords:** CCL21, CCR7, MEK-ERK, axonal growth, nociception

## Abstract

While chemokines were originally described for their ability to induce cell migration, many studies show how these proteins also take part in many other cell functions, acting as adaptable messengers in the communication between a diversity of cell types. In the nervous system, chemokines participate both in physiological and pathological processes, and while their expression is often described on glial and immune cells, growing evidence describes the expression of chemokines and their receptors in neurons, highlighting their potential in auto- and paracrine signalling. In this study we analysed the role of nociception in the neuronal chemokinome, and in turn their role in axonal growth. We found that stimulating TRPV1^+^ nociceptors induces a transient increase in CCL21. Interestingly we also found that CCL21 enhances neurite growth of large diameter proprioceptors *in vitro*. Consistent with this, we show that proprioceptors express the CCL21 receptor CCR7, and a CCR7 neutralizing antibody dose-dependently attenuates CCL21-induced neurite outgrowth. Mechanistically, we found that CCL21 binds locally to its receptor CCR7 at the growth cone, activating the downstream MEK-ERK pathway, that in turn activates N-WASP, triggering actin filament ramification in the growth cone, resulting in increased axonal growth.

## Introduction

Classically, chemokines have been associated with leukocyte migration (1). Nevertheless, growing evidence shows that they can signal to a great variety of cell types and tissues, including neural cells (2, 3). In addition, as conventional chemokine receptors are G-protein coupled receptors (GPCRs), chemokines can initiate a broad variety of intracellular signaling pathways (1).

Several studies have demonstrated the presence and the importance of chemokines in the nervous system (2, 4, 5). For instance, CXCL12 plays a vital role in regulating neuronal migration during cortical development (6, 7). Remarkably, different studies show both chemokines and their receptors expressed in neurons, suggesting an implication of these components in direct neuronal communication (5, 8). In homeostasis, neuronal CX_3_CL1 interacts with microglia preventing its activation (9). Additionally, neuronal chemokines have emerged as fundamental signals after insult. For instance, CCL2 or CCL21 (10–13), are secreted upon neuronal injury and serve as chemoattractants of immune cells, triggering their activation. This activation, however, often leads to neuropathic pain (11–13). However, some chemokines, such as CCL2 after peripheral injury, have been also described to promote axonal regeneration as a result of macrophage recruitment and phenotype modulation (10, 14). Meanwhile, although several chemokine receptors are expressed in different neuronal types (8, 9), little is known about their functions as autocrine or paracrine messengers on other neurons.

Neurons alter their secretome when exposed to different stimuli and according to their physiological state. In that direction, neuronal activity has been shown to modulate neuronal communication, including with microglia or with other neurons beyond classical neurotransmission (15–17). Nociceptor activity after axonal injury is normally associated with pathological neuropathic pain (18), despite that, some studies uncover how nociception participates in the healing process, such as promoting skin or adipose tissue regeneration, as well as neovascularization (19–21). These findings indicate that nociception might function as a key component of the healing machinery, and it is therefore important to study its precise roles in healing and regeneration in different tissues, including the nervous system.

In that sense, injured nociceptors have been described to release CCL21, however, whether this expression affected axonal regeneration has not been previously assessed (13). CCL21 has a primordial role in immune cell homing, *via* its canonical receptor CCR7 (22), but other functions for this chemokine in distinct tissues are also emerging, such as cartilage regeneration (23) and neuropathic pain induction in the CNS (13). Interestingly, in accordance with its function as a migration cue, the CCL21-CCR7 interaction activates intracellular pathways related to chemotaxis, *via* ERK signaling, this includes actin cytoskeleton remodeling *via* RhoA (24). Since cellular migration and growth cone dynamics are analogous mechanisms (25, 26), we hypothesized that CCL21 could exert a growth promoting effect on neurons.

In the present study, we investigated the impact of nociceptor activation in the neuronal chemokinome which led us to find an undescribed mechanism of neuronal communication between two different neuronal types, nociceptors and proprioceptors. Specifically, we found that activation of TRPV1^+^ nociceptors induces an increase in CCL21 expression. Moreover, we revealed a novel role for this CCL21 in proprioceptors, promoting neurite outgrowth. We then found that the receptor CCR7, expressed in proprioceptors, was required for this effect, which was also dependent on the MEK-ERK pathway. Finally, our results disclose a local mechanism in the growth cone, where CCR7 expression is concentrated, affecting the actin cytoskeleton, ultimately leading to enhanced neurite outgrowth.

## Materials and Methods

### Mice

B6.Cg-Tg(Thy1-COP4/EYFP)18Gfng/J (Thy1-ChR2) (27) were obtained from Jackson Laboratories and C57BL/6J mice from Charles River Laboratories. PV-Cre/Ai27D/CSP-Flox mice (#008069 and #012567 (Jackson Laboratories) crossbreeding) were provided by Dr. Rafael Fernández Chacón (Instituto de Biomedicina de Sevilla, Spain). Mice ranging from 6-10 weeks were used. All animal work was approved by the Ethics Committee on Animal Experimentation (CEEA) of the University of Barcelona (procedures #276/16, 47/20, and OB41/21).

### Compounds

Capsaicin (Merck, 100μg/ml i.pl.), CCL21 (Peprotech, 100ng/sciatic nerve *ex vivo*; 1nM, 10nM, 50nM *in vitro*);, αCCR7 blocking antibody (R&D systems, 2-5μg/ml), rat immunoglobulin (Ig) G (Merck, 2-5μg/ml), U0126 (Promega, 1μM), Wiskostatin (Merck, 1 μM), CCL19 (Peprotech, 1 nM, 10nM, 50nM), Pertussis toxin (Merck, 50ng/ml).

### *In Vitro* Optogenetic Stimulation

Thy1-ChR2 DRG neurons were used for *in vitro* optogenetic stimulation. A 470nm emission LED array (LuxeonRebelTM) under the control of a Driver LED (FemtoBuck, SparkFun) of 600mA and a pulse generator PulsePal (28) were used to deliver blue light to neuronal cultures. The optogenetic stimulation protocol consisted in 1h of illumination at 10Hz of frequency with 10ms-90ms pulses, in 1s ON-4s OFF periods. Stimulation was applied 2h after seeding.

### Chemokinome Quantification

A Mouse Chemokine Array C1 (RayBiotech) was used to determine the expression levels of 25 different chemokines in the media of cultured optogenetically stimulated and non-stimulated DRGs. The media was recovered 24h after the stimulation and processed as indicated by the manufacturer.

Quantificatrion was performed by densitometry using Gilles Carpentier's Dot-Blot-Analyzer macro [written by Gilles Carpentier, 2008 (29)]. The macro is available at http://rsb.info.nih.gov/ij/macros/toolsets/Dot%20Blot%20Analyzer.txt and more information can be found at http://image.bio.methods.free.fr/dotblot.html) written for ImageJ. The intensity measurements of the different chemokines were normalized to a reference spot and to the non-stimulated samples.

### Capsaicin Administration

Intraplantar (i.pl.) injection of capsaicin (10μl of 100μg/ml in PBS + 10% ethanol) was performed 2h before animal sacrifice and DRG dissection.

### Dorsal Root Ganglia Neuronal Culture

DRGs were dissected, collected in ice-cold Hank’s balanced salt solution (HBSS) (ThermoFisher Scientific) and transferred to a digestion solution [5mg/ml Dispase II (Merck) and 2.5mg/ml Collagenase Type II (ThermoFisher Scientific) in DMEM (ThermoFisher Scientific)] incubated for 45min at 37°C. DRGs were then centrifuged, and digestion solution was then exchanged for DMEM:F12 (ThermoFisher Scientific) media supplemented with 10% heat inactivated FBS and 1x B27 (ThermoFisher Scientific), the DRGs were then dissociated by pipetting. The resulting cell suspension was then spun down, resuspended in culture media (DMEM:F-12 media supplemented with 1x B27 and penicillin/streptomycin (P/S) (ThermoFisher Scientific)) and seeded in 48-well plates (3000-4000 cells/well) previously coated with 0.1mg/ml poly-D-lysine (2h, 37°C; Merck) and 2μg/ml laminin (over-night (O/N), RT (room temperature); ThermoFisher Scientific). Cells were kept at 37°C in a 5% CO_2_ atmosphere.

Compounds were added 2h after plating, unless in combinatorial experiments, when blocking antibodies or pharmacological inhibitors were added at 1,5h and 50nM of CCL21 30 min later. All compounds were left in the media for the whole duration of the experiment. Cells were fixed 24h after the treatment.

### Local Administration to the Sciatic Nerve

Mice were anaesthetized with inhaled isofluorane (4% induction, 2% maintenance). The sciatic nerve was hooked and immobilized after skin incision and blunt dissection of the gluteus maximus and the biceps femoralis. Compounds were locally injected into the nerve and the incision was closed by layer. Animals were then allowed to recover for 24h.

### *Ex Vivo* DRG Culture

2h after capsaicin i.pl. administration or 24h after local administration of CCL21 to the sciatic nerve respectively, sciatic DRGs (L4, L5, L6) were dissected and processed for cell culture as previously explained. DRGs from each animal were plated separately, and each experimental group was plated equally and did not receive any further treatment.

### Immunocytochemistry

Cells were fixed with cooled 4% paraformaldehyde (PFA) for 15min and washed in PBS (0.1M). The cells were then blocked [PBS-0.25% Triton X-100+ 1% BSA (bovine serum albumin)] for 1h at RT and then primary antibodies (anti‐βIII tubulin (Tuj1, 1:1000, BioLegend), anti-NFH (1:1000, Merck), anti-CCR7 (1:200, Abcam; ab32527 (30))), were incubated O/N at 4°C in the same solution. After several washes, Alexa-Fluor-conjugated secondary antibodies were incubated in blocking solution for 1h at RT and cell nuclei were counterstained with Hoechst.

### Immunohistochemistry

DRGs were dissected and fixed in PFA 4% for 2h at 4°C. After washing the DRGs were transferred into cryoprotection solution (PBS-30% sucrose) for 24h at 4°C. Tissue freezing medium (OCT, Merck) was used to freeze the DRGs in blocks, and then 10μm slices were generated with a cryostat (Leica CM 1900) and mounted on slides. The slides were washed in TTBS (TBS (Tris-buffered saline) + 1% Tween) and blocking solution (8% BSA and 0.3% Tx and 1/150 mαIgG (Jackson Immuno Research)) was then incubated for 1h at RT. After an O/N incubation with primary antibodies (anti-TRPV/VR1 (1:200, Santa Cruz; sc-398417 (31)), anti-CCL21 (1:200 Peprotech; 500-P114 (32)), anti-CCR7 [1:200, Abcam; ab32527 (30))] in 2% BSA and 0.2% Tx in TBS at 4°C, and washing, Alexa-Fluor-conjugated secondary antibodies were added in the same solution for 1h at RT. Preparations were then mounted with coverslips in Mowiol™ (Merck).

### Western Blot

DRG neurons were seeded in 24-well plates (10.000 cells/well) and treated with 50nM of CCL21 2h after seeding. 50min after the treatment media was aspirated and the cells were lysed in 40µl of sample buffer [0.25M Tris HCL (Sigma-Aldrich), 4%SDS (Sigma-Aldrich), 40% Glycerol (Sigma-Aldrich)] + 10% of β-mercaptoethanol (ThermoFisher Scientific) and detached using a scraper. Cell lysates were then heated at 95°C for 10 min and 10 µl were loaded in SDS-10% polyacrylamide (Bio-Rad) gel electrophoresis (PAGE) gels. The gels were transferred to nitrocellulose membranes (Merck) for 1h at 4°C and the membranes were blocked for 1h with 3% BSA-TTBS at RT and O/N at 4°C with primary antibody (pERK (1:1000, Cell Signaling) or 2h at 37°C [GAPDH (1:10.000, ThermoFisher Scientific)]. After several washes, Horseradish peroxidase (HRP)-linked secondary antibodies (Dako) were added to the membranes and incubated for 1h at RT, and finally developed with ECL™ substrate (Merck). Intensity quantifications were performed using ImageJ.

### Fluorescence Intensity Analysis

DRG slices were stained for CCL21 and TRPV1. Images were acquired with a LSM 800 confocal microscope (Zeiss) using an AxioCam 503c camera (Zeiss) at 20X magnification. Mean CCL21 fluorescence intensity (MFI) was computed in TRPV1^+^ cells using ImageJ, subtracting the background for each image.

### Neurite Length Analysis

Tuj1 immunocytochemistry was performed and imaged at 10X magnification using an Olympus microscope IX71 with an Orca Flash 4 (3 images per well). The neurite length of large-diameter (>35μm) neurons was blindly quantified with the Neuron J plugin for ImageJ (33). Average neurite length per neuron was computed.

### Statistical Analysis

Statistics and graphical representation were carried out using Prism 6.0 (GraphPad™ Software). Shapiro-Wilk test was used to verify normality of the distributions. * or # indicate significant differences in ANOVA or Kruskall Wallis followed by Bonferroni’s, Dunn’s *post-hoc* test or Student’s t-test. Plotted data represents mean ± s.e.m (standard error of the mean). All tests performed were two-sided, and adjustments for multiple comparisons and/or significantly different variances (Fisher’s F) were applied were indicated. All data analysis was performed blind to the experimental group by two independent experimenters. Unless otherwise stated, sample size was chosen in order to ensure a power of at least 0.8, with a type I error threshold of 0.05, in view of the minimum effect size that was expected.

## Results

### CCL21 Expression is Upregulated Upon Nociceptor Activation

**Figure 1:**
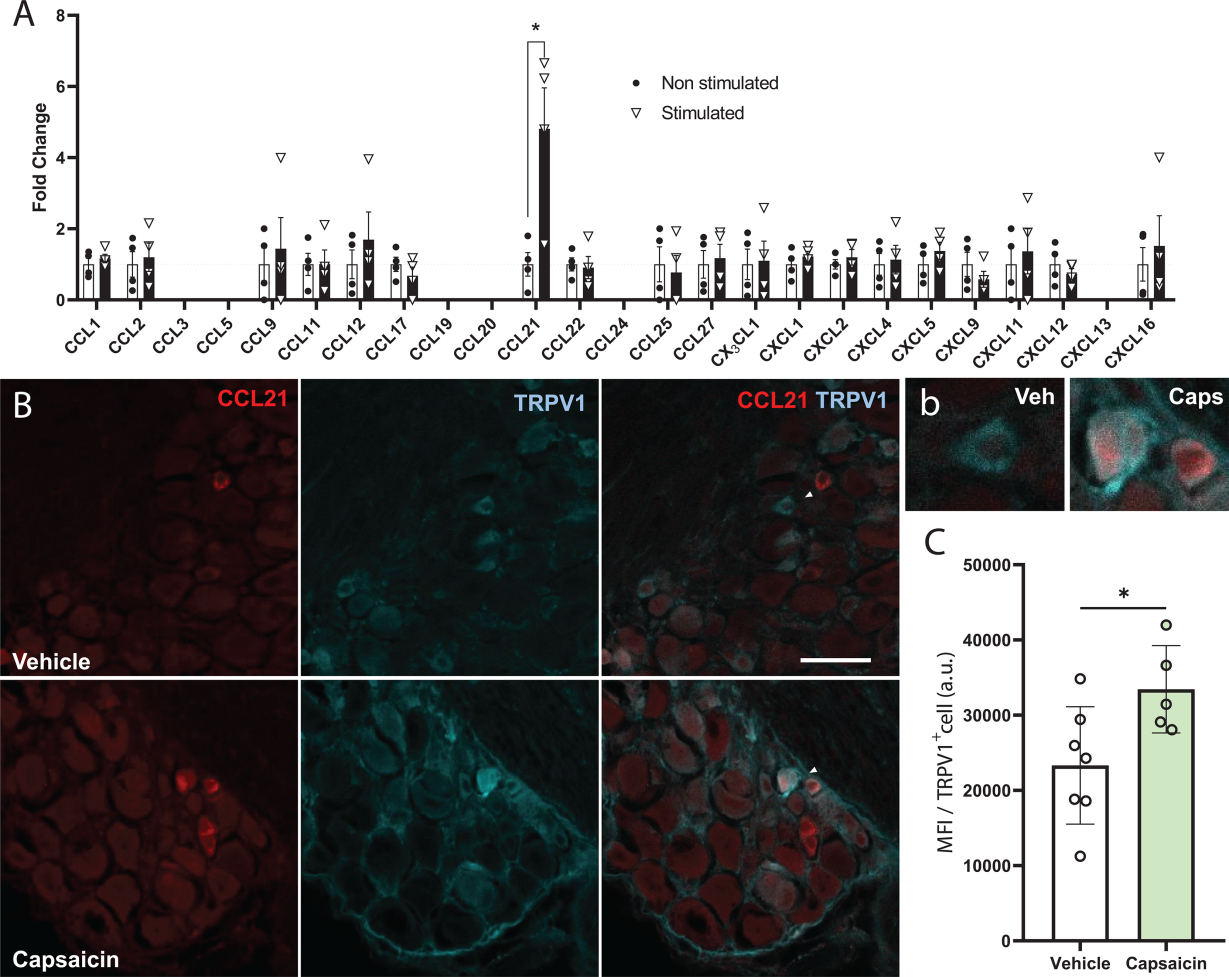
CCL21 is upregulated upon TRPV1^+^ nociceptive neuron activation. **(A)** Chemokine array data shows that only CCL21 expression was increased after optogenetic stimulation. CCL3, CCL5, CCL19, CCL24 and CXCL13 were not detected. Data are expressed as mean fold change of normalized array spots intensity ± s.e.m, Multiple Student’s t-test; *p < 0.05 (n=4). **(B)** Immunohistochemistry showing CCL21 expression in TRPV1^+^ neurons 2h after vehicle or capsaicin i.pl. injection. White arrows: magnified neurons in **(b)** Scale bar: 50μm. High magnification inset. **(C)** Intraplantar capsaicin injection increased CCL21 expression in TRPV1^+^ nociceptors specifically as shown by mean fluorescence intensity (MFI) of TRPV1^+^ cells. a.u., arbitrary units. Data are expressed as mean ± s.e.m; Student’s t-test; *p < 0.05; n=5-7 images.

While most defined neuronal chemokines are induced after traumatic injury or inflammatory signalling (5), we analysed whether stimulating neuronal activity in the DRG would have an impact in chemokine expression and secretion. To this aim we used DRG neuronal cultures expressing ChR2 (from Thy1-ChR2 animals) and subjected them to optical stimulation. We recovered the media 24h later and measured chemokine secretion using a Mouse Chemokine Array (Raybiotech). Interestingly, we found a remarkable increase in CCL21 levels compared to non-stimulated controls (**Figure 1A**). ChR2 expression in DRGs is ubiquitous among all sensory subtypes (**Supplementary Figures 1A, B**), however, previous studies reported CCL21 expression specifically in small diameter TRPV1^+^ nociceptors after peripheral nerve injury (13, 34), so we hypothesized that this CCL21 increase after optogenetic stimulation could be specific of this neuronal subtype. Accordingly, CCL21 expression was increased in TRPV1^+^ nociceptors 2h after i.pl capsaicin (TRPV1 agonist) injection as compared to vehicle administered animals (Student’s T test *p*= 0.0347) (**Figures 1B, C**).

### CCL21 Promotes Neurite Outgrowth

**Figure 2:**
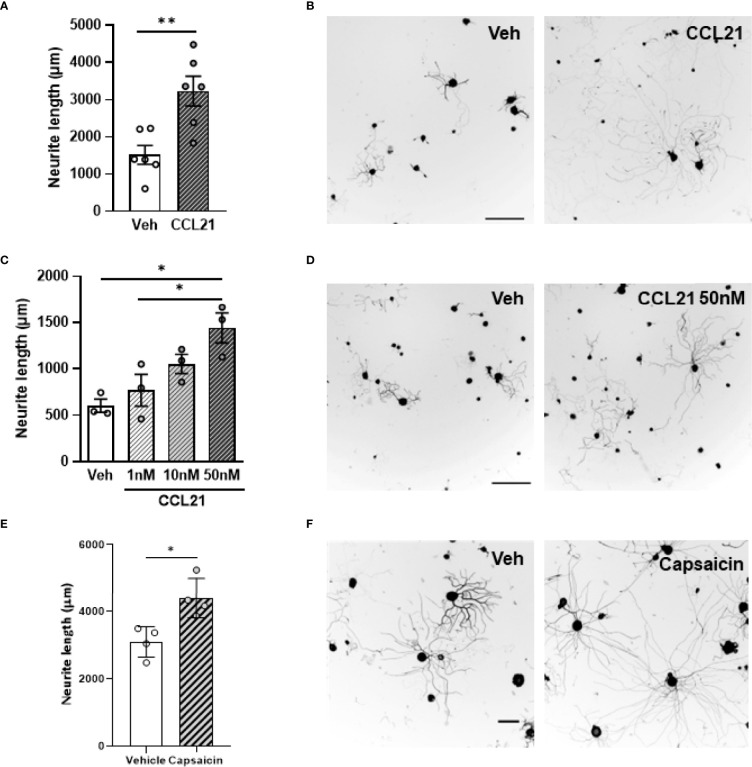
CCL21 enhances DRG neurite outgrowth. **(A)** *Ex vivo* CCL21 administration in the sciatic nerve promoted neurite outgrowth of DRG large-diameter neurons. Data are expressed as average neurite length per neuron ± s.e.m; Student’s t-test; **p < 0.01; n=6 sciatic nerves. **(C)** Dose-dependent increase in neurite length of *in vitro* CCL21-treated DRG large-diameter neurons. Data are expressed as average neurite length per neuron ± s.e.m; One-way ANOVA, Bonferroni’s *post-hoc*; *p < 0.05; n=3 wells. **(B, D)** Tuj-1 representative immunostainings **(B**: *ex vivo* **D**: *in vitro***)** after 24h in culture. Scale bar: 250μm. **(E)** Intraplantar capsaicin administration increased neurite outgrowth of *ex vivo* cultured DRG large-diameter neurons. Data are expressed as average neurite length per neuron ± s.e.m; Student’s t-test; *p < 0.05; n=4 biological replicates. **(F)** Tuj-1 representative immunostainings after 24h in culture. Scale bar: 100μm.

To test whether this chemokine could also influence axonal growth, we administered CCL21 into the sciatic nerve and cultured disaggregated DRG neurons 24h after. This led to an increment in the neurite outgrowth of *ex vivo* CCL21-treated DRG neurons when compared to vehicle treated ones (Student’s T test *p*= 0.0046) (**Figures 2A, B**). Additionally, administration of CCL21 at different doses (1nM, 10nM, 50nM) on DRG cultures, resulted in a dose-dependent increase of DRG neurite outgrowth *in vitro* (ANOVA followed by Bonferroni test; Veh vs 50nM *p*= 0.0129; 1nM vs 50nM *p*= 0.0440) (**Figures 2C, D**). To further prove that CCL21 produced by TRPV1^+^ nociceptors could also induce proprioceptive neurite outgrowth, we cultured disaggregated DRG neurons 2h after i.pl. capsaicin administration, a setting in which we observed an increase in CCL21 expression (**Figures 1B, C**), and observed an increased outgrowth as compared to vehicle treated animals (Student’s T test *p*= 0.0129) (**Figures 2E, F**).

### CCL21 Activates Proprioceptive CCR7 to Promote Neurite Outgrowth

**Figure 3:**
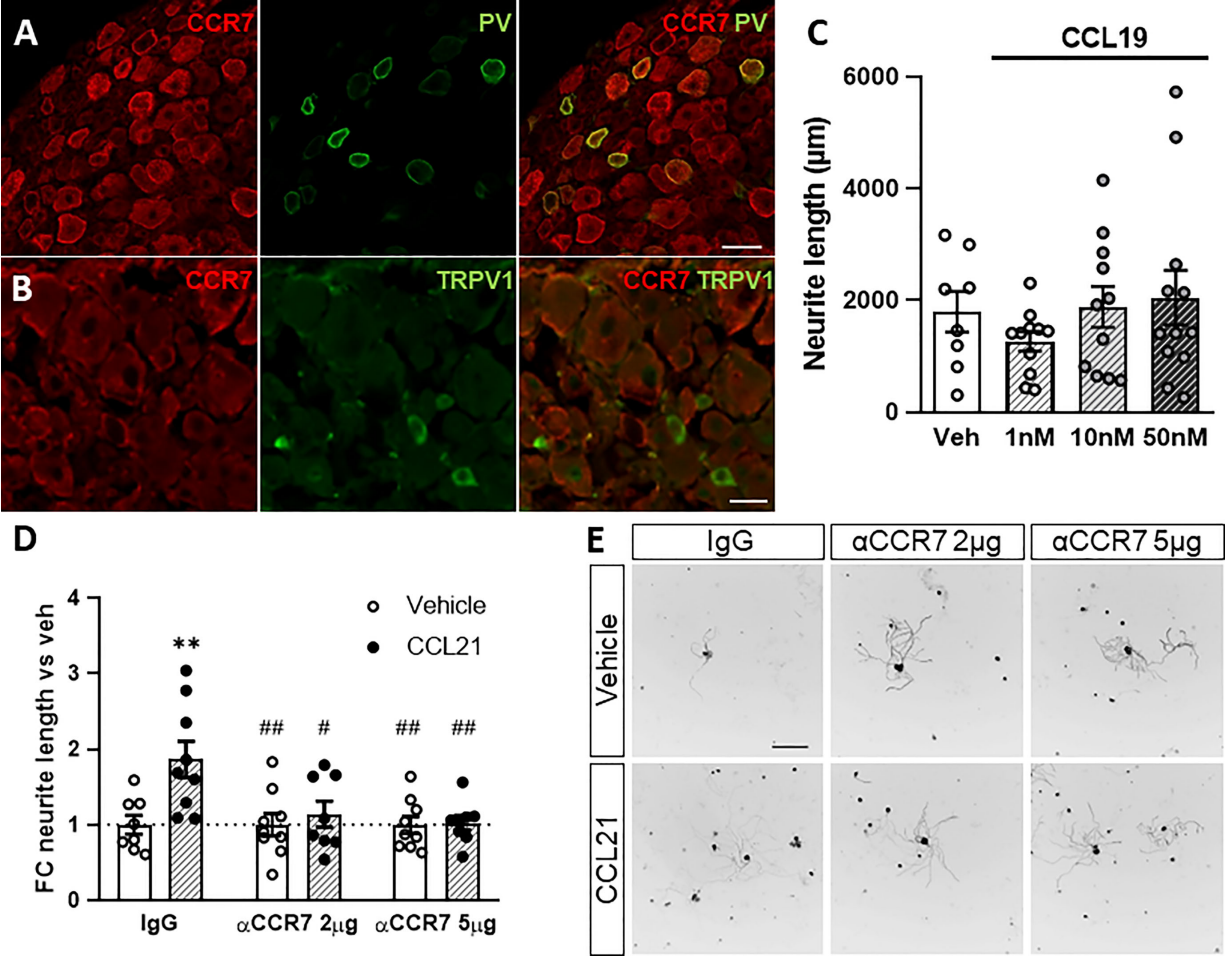
CCR7 is required for CCL21-mediated DRG outgrowth. A-B. PV^+^ but not TRPV1^+^ neurons express the canonical CCL21 receptor CCR7. Scale bar: 50μm **(A)** 25μm **(B)**. **(C)** CCL19 administration does not induce neurite outgrowth. Data are expressed as average neurite length per neuron ± s.e.m; n=8-12 images. **(D)** CCR7-blockade abolished the CCL21-dependent growth induction. Data are expressed as mean fold change of average neurite length per neuron vs each vehicle group ± s.e.m. Two-way ANOVA, Bonferroni’s *post-hoc*; **p < 0.01 (vs IgG-veh); #*p* < 0.05; ##*p* < 0.01 (vs IgG-CCL21); n=8-9 images. **(E)** Tuj-1 representative immunostainings after 24h in culture. Scale bar: 250μm.

CCL21 is a functional ligand of CCR7 (35). We therefore analysed the expression pattern of CCR7 in the DRG. We found CCR7 expression mainly in neurons, including in parvalbumin^+^ (PV) neurons, that correspond to proprioceptors (**Figure 3A**), and very low number of CCR7^+^TRPV1^+^ nociceptors (**Figure 3B**). Additionally, administering a CCR7-blocking antibody to DRG cultures inhibited the neurite outgrowth induced by CCL21 as compared to the IgG control (two-way ANOVA followed by Bonferroni test; interaction p= 0.0233; IgG-veh vs IgG-CCL21 p= 0.0055; IgG-CCL21 vs αCCR7 2μg-veh p= 0.0040; IgG-CCL21 vs αCCR7 2μg-CCL21 p= 0.0355; IgG-CCL21 vs αCCR7 5μg-veh p= 0.0037; IgG-CCL21 vs αCCR7 5μg-CCL21 p= 0.0079) (**Figures 3D, E**). Parallelly, although its expression is not detectable in DRGs (**Figure 1A**), we also tested whether CCL19, another CCR7 ligand (35), would induce the same effects. Oppositely, CCL19 did not result in increased neurite outgrowth when administered *in vitro* (Kruskal-Wallis test followed by Dunn’s test; Veh vs 50nM p > 0.9999) (**Figure 3C**), suggesting a CCL21 biased CCR7 activation is responsible for this particular mechanism.

### CCL21-CCR7 activates the MEK-ERK Pathway

**Figure 4:**
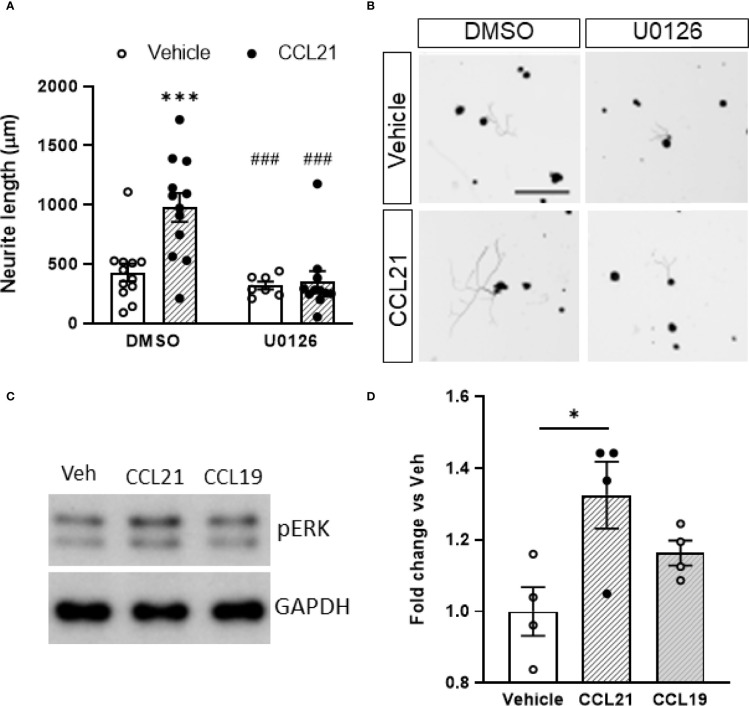
CCL21 induced outgrowth through biased activation of the MEK/ERK pathway. **(A)** CCL21 and U0126, a MEK inhibitor, co-administration resulted in reduced neurite outgrowth compared to CCL21 administration alone. Data are expressed as average neurite length per neuron ± s.e.m; Two-way ANOVA, Bonferroni’s *post-hoc*; ***p < 0.001 (vs veh-DMSO), ###p < 0.001 (vs CCL21-DMSO). n=7-12 images. **(B)** Tuj-1 representative immunostainings after 24h in culture. Scale bar: 250μm. **(C)** Representative Western Blot images showing pERK expression in CCL21 and CCL19-treated DRG cultures. **(D)** pERK expression is increased 50 minutes after the administration of CCL21, while the administration of CCL19 shows a reduced pERK induction. Data are expressed as mean ± s.e.m; One-way ANOVA, Bonferroni’s *post-hoc*; *p < 0.05; n=4.

We then targeted the two main known downstream actuators of CCR7 activation, the MEK pathway and the G_i/o_ protein previously known to be involved in axon growth (36–38). Pertussis toxin (Ptx) administration, an inhibitor of the G_i/o_ protein, did not prevent the CCL21-induced outgrowth (**Supplementary Figure 2**) as evidenced by the lack of significant interaction of the treatments (*p*= 0.7961) on the two-way ANOVA (Student’s t-test w/o (without) Ptx: *p*= 0.0115; with Ptx: *p*= 0.7458). Conversely, pharmacological inhibition of MEK with U0126 blocked the neurite outgrowth induced by CCL21 (**Figures 4A, B**), as evidenced by the significant interaction of the treatments (*p*= 0.0117) on the two-way ANOVA (multiple comparisons with Bonferroni test: DMSO-Veh vs DMSO-CCL21 *p*= 0.0005; DMSO-CCL21 vs U0126-Veh *p*= 0.0004; DMSO-CCL21 vs U0126-CCL21 *p*= 0.0001). Additionally CCL21 but not CCL19 induced an increase in the phosphorylation of ERK (**Figures 4C, D**), proving at the same time the presence of a biased CCL21-CCR7 signalling as well as the role of the MEK-ERK pathway in the CCL21-CCR7 induced neurite growth.

### CCL21-CCR7-MEK Pathway Modulates Actin Cytoskeleton to Promote Neurite Outgrowth

**Figure 5:**
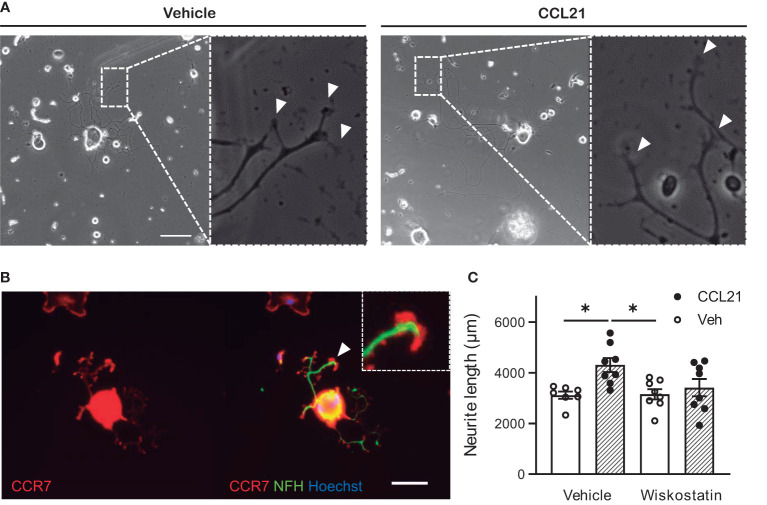
CCL21 stimulates actin dynamics in the growth cone. **(A)** CCL21 administration resulted in enlarged growth cones in large-diameter DRG neurons. White arrows: growth cones. Scale bar: 100μm. **(B)** CCR7 expression is specially elevated in the growth cone. Scale bar: 50μm. **(C)** Actin branching is important for CCL21-dependent neurite outgrowth, as shown by impaired growth after wiskostatin treatment. Data are expressed as average neurite length per neuron ± s.e.m; One-way ANOVA, Bonferroni’s *post-hoc*; *p < 0.05; n=7-8 wells.

Local assessment of the axonal tips revealed larger growth cones after CCL21 treatment (**Figure 5A**), this is in consonance with the especially abundant CCR7 expression found on these structures (**Figure 5B**). We then sought to check the local effects that CCL21 could have in cytoskeletal dynamics of the growth cones. Consequently, we co-administered CCL21 with a low dose of wiskostatin, an inhibitor of the neural Wiskott-Aldrich syndrome protein (N-WASP), that acts as an Arp2/3 complex activator. Wiskostatin greatly reduced the CCL21-induced growth, while not having an effect by itself (**Figure 5C**), supporting a local effect in actin dynamics and branching by CCL21 in the growth cone (two-way ANOVA followed by Bonferroni test; interaction *p*= 0.0825; one-way ANOVA followed by Bonferroni test; DMSO-Veh vs DMSO-CCL21 *p*= 0.0207; DMSO-CCL21 vs Wiskostatin-Veh *p*= 0.0202).

## Discussion

There are plenty of evidence that neuronal activity can alter neuronal signalling. In that sense, we found that the expression and release of the chemokine CCL21 is enhanced upon neuronal stimulation. Interestingly, we found that specific TRPV1^+^ nociceptor stimulation induces CCL21 production, similarly to what has already been described upon axonal injury (13).

After axonal injury, release of neuronal CCL21 is mainly linked to neuropathic pain (13, 39), however, its role in axon growth and regeneration had never been described before. Interestingly, our results revealed that CCL21 induces growth in proprioceptive neurons through activation of the CCR7-MEK-ERK pathway, exerting an effect on actin dynamics at the growth cone level.

CCL21 was first designated as a recruiting cue for leucocytes, specifically stimulating the migration of T cell subpopulations and dendritic cells (22, 40–42). More recently, it has also been described to induce migration in other cells such as tumorigenic or mesenchymal stem cells (23, 43–46). Fundamentally, cell migration and axonal growth are events that share similar cellular and molecular machinery (25, 26). Thus, molecules that orchestrate one process are or will most likely be implicated in the other, and vice versa, as for instance what occurs with CXCL12 (6, 47–49).

We also describe that CCL21 executes its growth-inducing function through its canonical receptor CCR7, in accordance, we found abundant expression of this receptor on proprioceptive (PV^+^) neurons, both on the soma and on the axon, similarly to the findings of other studies, where neuronal CCR7 is abundantly found in peripheral nerves and hippocampal neurons (50, 51). Activation of CCR7 has already been shown to play a cardinal role in the CCL21-induced cell migration (26, 40, 43, 45, 52), that, as stated, activates similar cellular and molecular machinery than axonal growth (25, 26).

We also found that pharmacological inhibition of the MEK-ERK pathway, one of the main downstream mediators of CCR7 effects, inhibited CCL21-dependent outgrowth. Additionally, CCL21 administration induced phosphorylation of ERK. These results are in line with other studies defining MEK-ERK as the underlying mechanism of the chemotaxis induced by CCL21-CCR7 (40, 53). Contrarily, we did not see an effect by inhibiting the G_i/o_ protein in the CCL21- induced outgrowth, however lack of growth-suppression could derive from inactivation of other growth-inhibitor pathways activated by the G_i/o_ (54).

While we did not observe any DRG expression of CCL19, we wanted to test if the other canonical ligand of CCR7 would elicit the same effects observed with CCL21. Intriguingly, CCL19 administration, did not induce neither ERK phosphorylation nor axonal growth *in vitro*. These findings strengthen the view of biased ligand-receptor responses, as already shown for CCL21 and CCL19, which can trigger specific downstream effectors of CCR7 (55, 56). These biased activations result in particular mechanisms activated only by CCL21, and not CCL19; however, this effect often varies depending on the target cell (57–60) MEK-ERK pathway activation has been previously implicated in axonal regeneration (37, 38, 61). For instance, after peripheral conditioning injury, ERK is phosphorylated and has been shown to affect multiple cellular processes affecting axonal growth, including transcriptional and epigenetic alterations, resulting in increased expression of several regeneration associated genes (RAGs) (61), increased retrograde transport (37, 62) as well as stimulation of cytoskeleton dynamics (38). According with the latter, ERK has been shown to have a direct effect on actin polymerization (63, 64), through phosphorylation of different effectors such as WAVE2, cortactin and Rac1 (65–69). Remarkably, we found CCR7 expression to be particularly elevated in the growth cones, therefore, we evaluated the effects of actin dynamics in the growth cone as a putative mechanism of the CCL21-induced growth. In that sense, coadministration of an N-WASP pharmacological inhibitor, a key actin polymerization component, blocked the growth induced by CCL21. N-WASP is an Arp2/3 complex activator, which in turn works as an actin-binding protein, mainly responsible for actin filament branching (70). Previous studies already showed the essential role of Arp2/3 in growth cone progression (71, 72). Mechanistically, ERK is known to phosphorylate cortactin, leading to N-WASP binding and activation (66).

**Figure 6:**
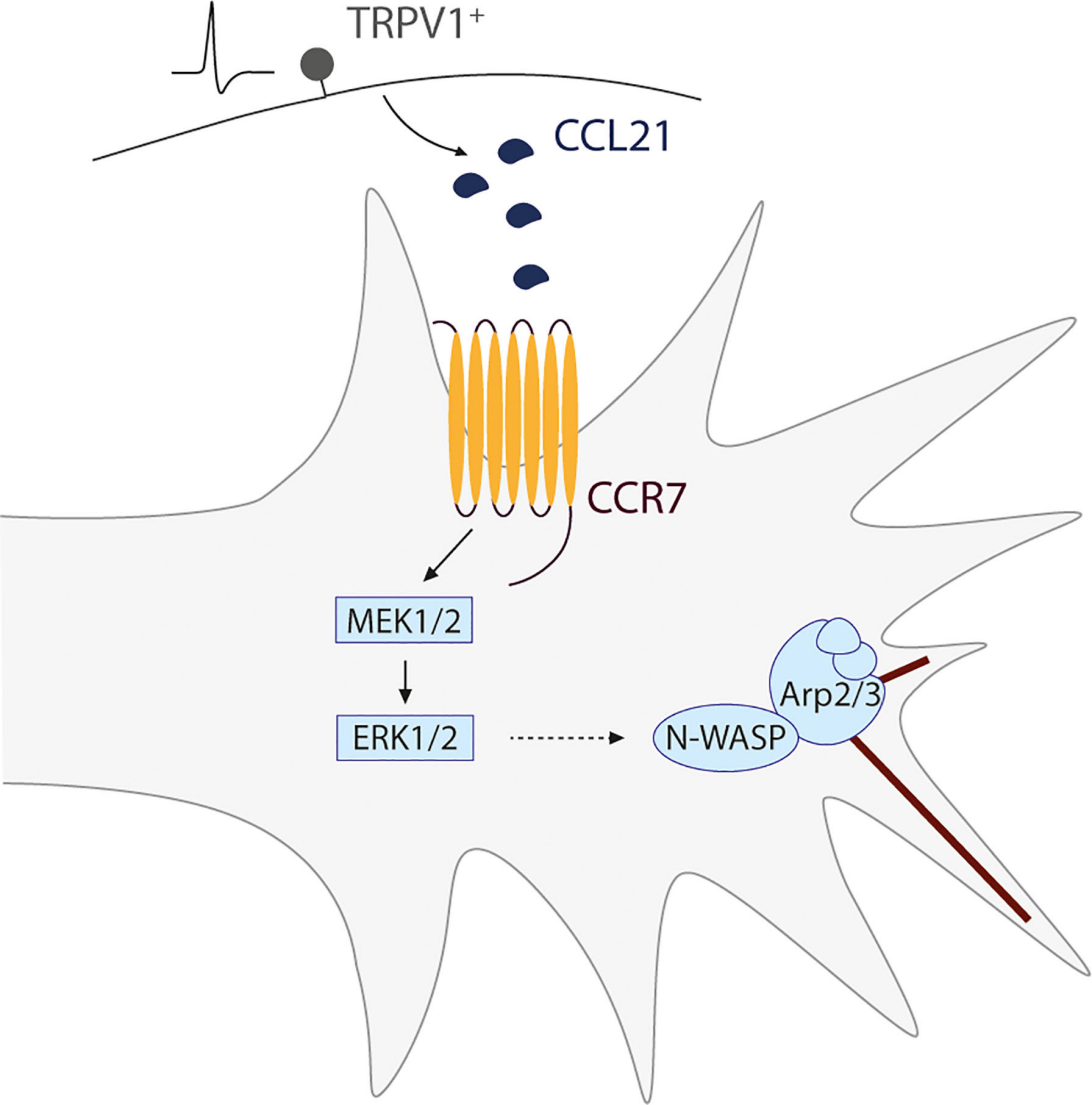
Schematic representation of the proposed mechanism for a novel nociceptor-proprioceptor dialogue leading to neuritic growth. Activated TRPV1^+^ nociceptors secrete CCL21 which promotes actin branching in the growth cone of proprioceptor neurons. This mechanism is mediated by the CCL21-CCR7 interaction, leading to a downstream activation of the MEK-ERK pathway and final N-WASP-related actin cytoskeleton modifications.

Thus, in a nutshell, we found that upon CCL21 binding to its receptor CCR7 in the axonal growth cone, there is a downstream MEK-ERK activation, that leads to N-WASP activation, triggering actin filament branching in this structure, resulting in increased axonal growth (**Figure 6**). Additionally, our *ex vivo* experiments, in which both capsaicin or CCL21 were administered in the animal before extracting the DRGs and culturing them, demonstrate a lasting effect on DRG somas leading to enhanced neurite outgrowth. While we cannot exclude the presence of a transcriptional reprogramming after CCL21-CCR7 activation, our model points towards a sustained effect on cytoskeletal remodelling induced by the increased MEK/ERK pathway activation.

While CCL21 has been already described for its role in regeneration in other tissues including skin, cartilage, and vascular remodelling (23, 44, 73), here we describe a novel role of this chemokine in axonal growth.

Additionally, the mechanism described involves an unprecedented paracrine dialogue based on chemokines between two different DRG neuronal types. While neurons are highly interconnected through synapses, there is little work regarding other forms of neuronal communication (74). Growth factors, for instance, have been implicated in both paracrine and autocrine neuronal communication (75–78), however, we describe a novel chemokine dependant mechanism of neuron-neuron signalling.

Parallelly, we have also unravelled a novel role of nociceptive signalling in sensory axon regeneration. Nociception and pain are tightly associated with tissue injury (79, 80), and while it is not surprising that a signal initiated by an injury could trigger the healing process, after nerve injury chemokine release and nociceptor activity have been typically linked to pathological neuropathic pain. Contrarily, here we show that stimulating nociceptor activity triggers the secretion of a growth-promoting chemokine, CCL21. In agreement with this, nociceptive signalling participates in the healing cascade of several tissues, for example, nociceptor activation induces adipose tissue regeneration through CGRP (calcitonin gene-related peptide) secretion (20), angiogenesis *via* Substance-P-mediated effects (19), and skin regeneration through modulation of the immune response (21). Pain and nociceptive signalling are complex evolutionary mechanisms that might have further implications than previously anticipated, as they play central roles orchestrating and promoting the healing process in different tissues.

This also suggests caution in the indiscriminate use of analgesic drugs and treatments after injury, as these may hinder nociceptive regenerative signalling, limiting the healing process, similarly to the effects observed by broad immunosuppressive drugs (81) or antioxidants (82) after spinal cord injuries. Therefore, appropriate timing and level of analgesic administration after injury will likely need to be tailored to provide pain relief while avoiding unwanted effects in hindering the tissue regeneration. An additional intriguing implication is the possibility to modulate nociceptive signalling to achieve tissue regeneration. While therapeutically, inducing nociception is not a reasonable approach, further investigation and characterization of signalling elicited by nociceptor stimulation may increase our understanding of the molecular mechanisms underlying the healing process, and may enable the future design of therapeutic targets and strategies to foster tissue regeneration.

## Data Availability Statement

The raw data supporting the conclusions of this article will be made available by the authors, without undue reservation.

## Ethics Statement

The animal study was reviewed and approved by Ethics Committee on Animal Experimentation (CEEA) of the University of Barcelona [Adolf Florensa, 8 p.1 office F14 08028 Barcelona (Spain)].

## Author Contributions

FM-V performed, designed experiments, performed data analysis, and wrote the manuscript. SM-T performed and designed experiments and performed data analysis. JD supervised experiments, provided experimental funds and edited the manuscript. AH performed, designed experiments, performed data analysis, provided experimental funds and wrote the manuscript. All authors contributed to the article and approved the submitted version.

## Funding

This research was supported by HDAC3-EAE-SCI Project with ref. PID2020-119769RA-I00 from MCIN/AEI/10.13039/501100011033 to AH and PRPSEM Project with ref. RTI2018-099773-B-I00 from MCINN/AEI/10.13039/501100011033/FEDER “Una manera de hacer Europa”, the CERCA Programme, and the Commission for Universities and Research of the Department of Innovation, Universities, and Enterprise of the Generalitat de Catalunya (SGR2017-648) to JD. The project leading to these results received funding from the María de Maeztu Unit of Excellence (Institute of Neurosciences, University of Barcelona) MDM-2017-0729 and Severo Ochoa Unit of Excellence (Institute of Bioengineering of Catalonia) CEX2018-000789-S from MCIN/AEI/10.13039/501100011033. FMV was supported by a fellowship from the “Ayudas para la Formación de Profesorado Universitario” (FPU16/03992) program, from the Spanish Ministry of Universities.

## Conflict of Interest

The authors declare that the research was conducted in the absence of any commercial or financial relationships that could be construed as a potential conflict of interest.

## Publisher’s Note

All claims expressed in this article are solely those of the authors and do not necessarily represent those of their affiliated organizations, or those of the publisher, the editors and the reviewers. Any product that may be evaluated in this article, or claim that may be made by its manufacturer, is not guaranteed or endorsed by the publisher.

## Supplementary Material

Supplementary Figure 1 ChR2 expression in Thy1-ChR2 DRGs. **(A)** Graph showing the proportion of ChR2^+^ cells depending on the neuronal diameter. ChR2 is expressed in both large-diameter (>35μm) and small-diameter (35-15μm) neurons. **(B)** ChR2 expression in Thy1-ChR2 DRGs. Scale bar: 50μm.

Supplementary Figure 2 Pertussis toxin addition does not prevent the CCL21-dependent neurite outgrowth increase. Data are expressed as mean fold change of average neurite length per neuron vs each vehicle ± s.e.m; n=9-12 images.
