# Supplementary figures and images for: Nociception-Dependent CCL21 Induces Dorsal Root Ganglia Axonal Growth via CCR7-ERK Activation
Source: Front Immunol. 2022 Jul 14;13:880647. doi: 10.3389/fimmu.2022.880647 (PMC9331658; doi:10.3389/fimmu.2022.880647)

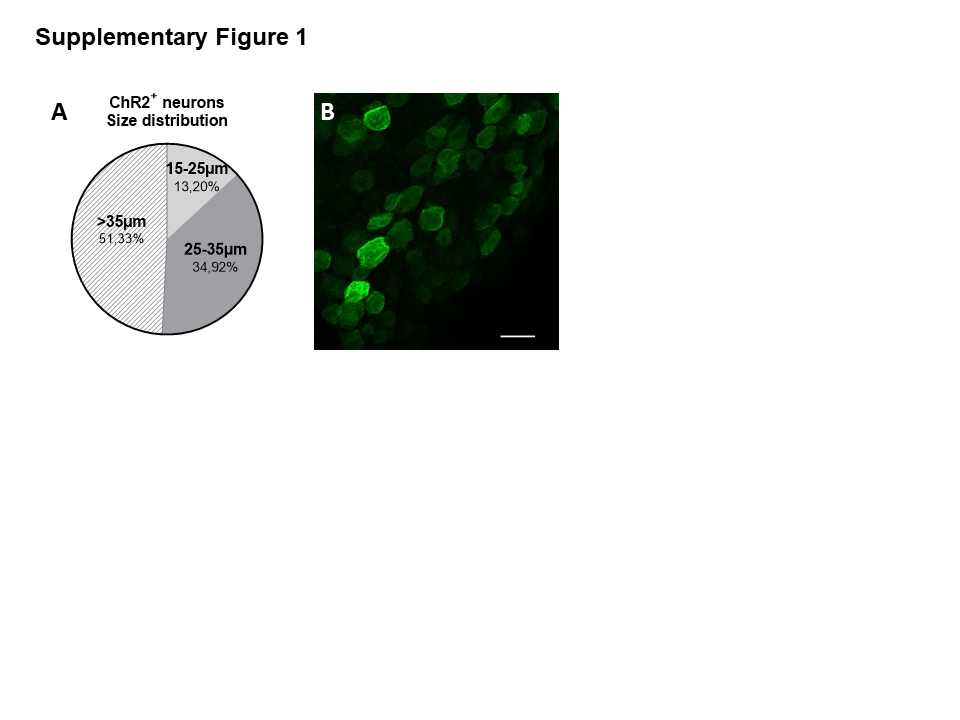

Supplement: Supplementary Figure 1 — ChR2 expression in Thy1-ChR2 DRGs. (A) Graph showing the proportion of ChR2+ cells depending on the neuronal diameter. ChR2 is expressed in both large-diameter (>35μm) and small-diameter (35-15μm) neurons. (B) ChR2 expression in Thy1-ChR2 DRGs. Scale bar: 50μm. [file Image_1.tif]

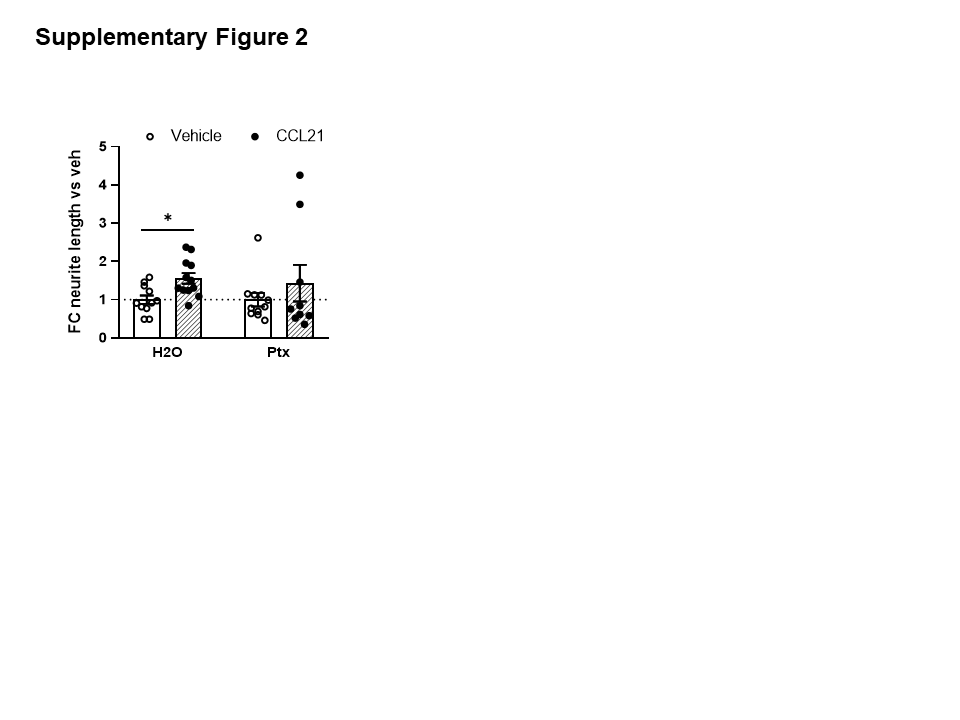

Supplement: Supplementary Figure 2 — Pertussis toxin addition does not prevent the CCL21-dependent neurite outgrowth increase. Data are expressed as mean fold change of average neurite length per neuron vs each vehicle ± s.e.m; n=9-12 images. [file Image_2.tif]
